# Supplementary figures and images for: Inheritance of Secondary Metabolites and Gene Expression Related to Tomato Fruit Quality
Source: Int J Mol Sci. 2022 May 31;23(11):6163. doi: 10.3390/ijms23116163 (PMC9181508; doi:10.3390/ijms23116163)

## Slide 1
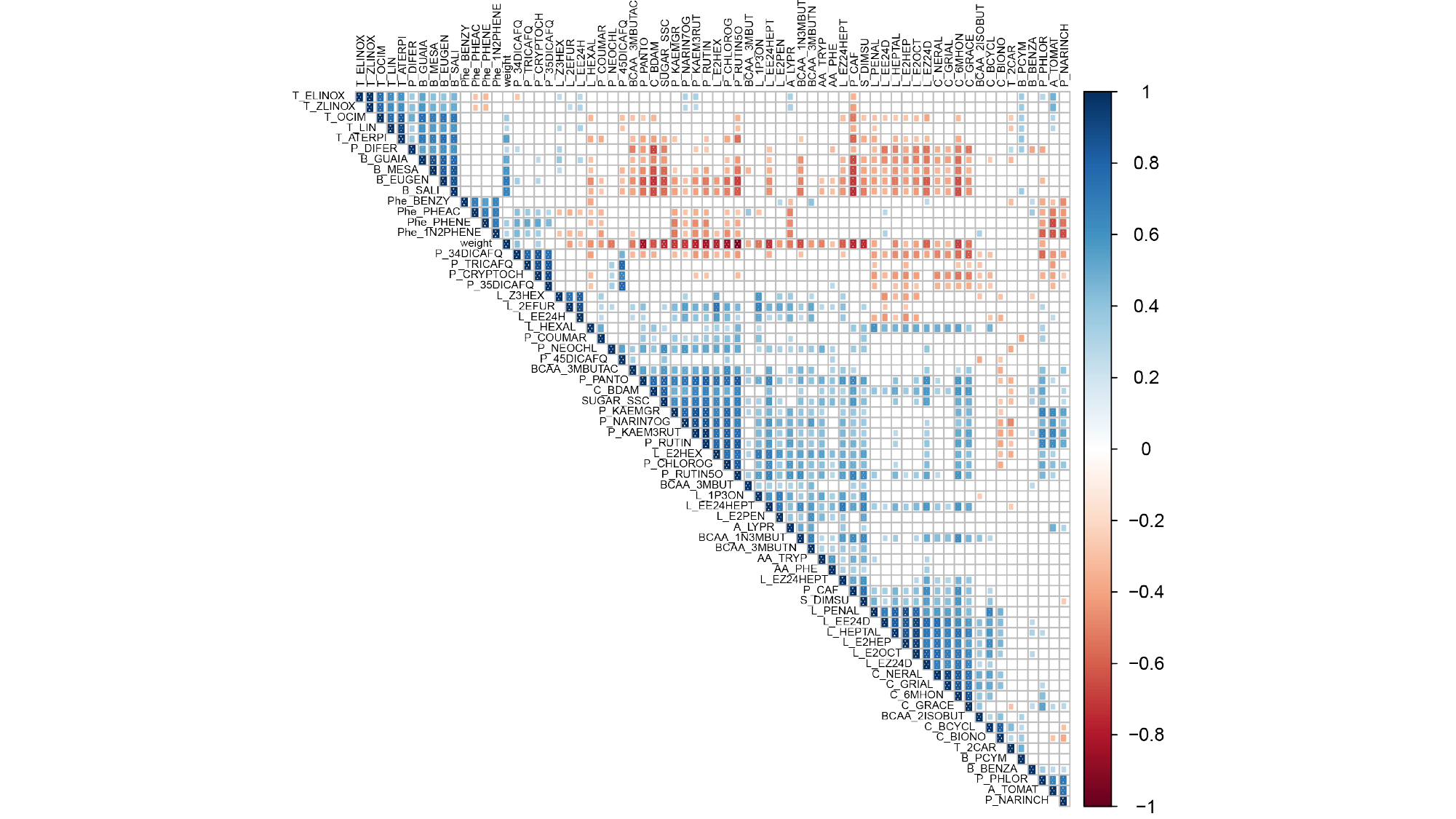

## Slide 2
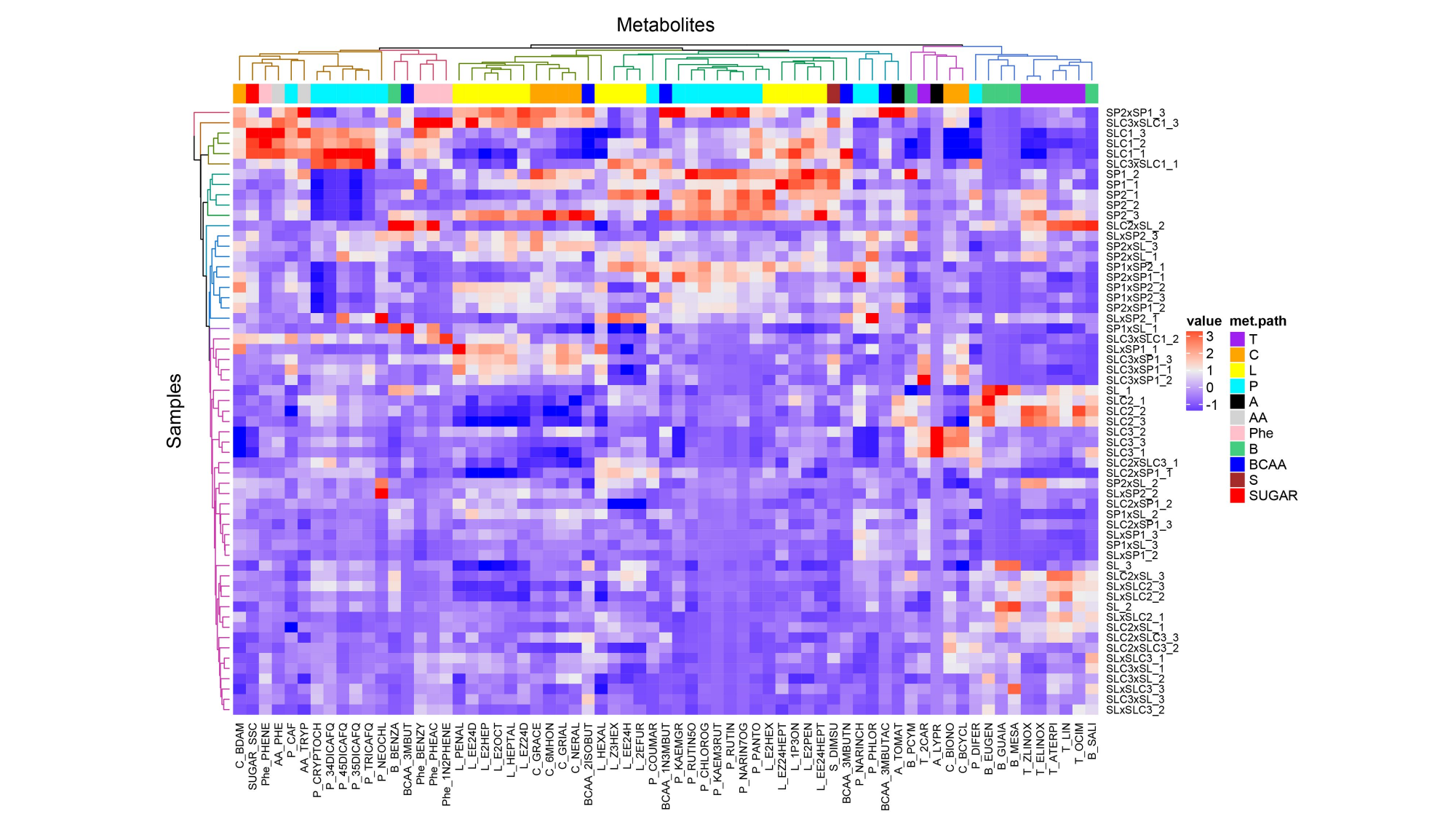

## Slide 3
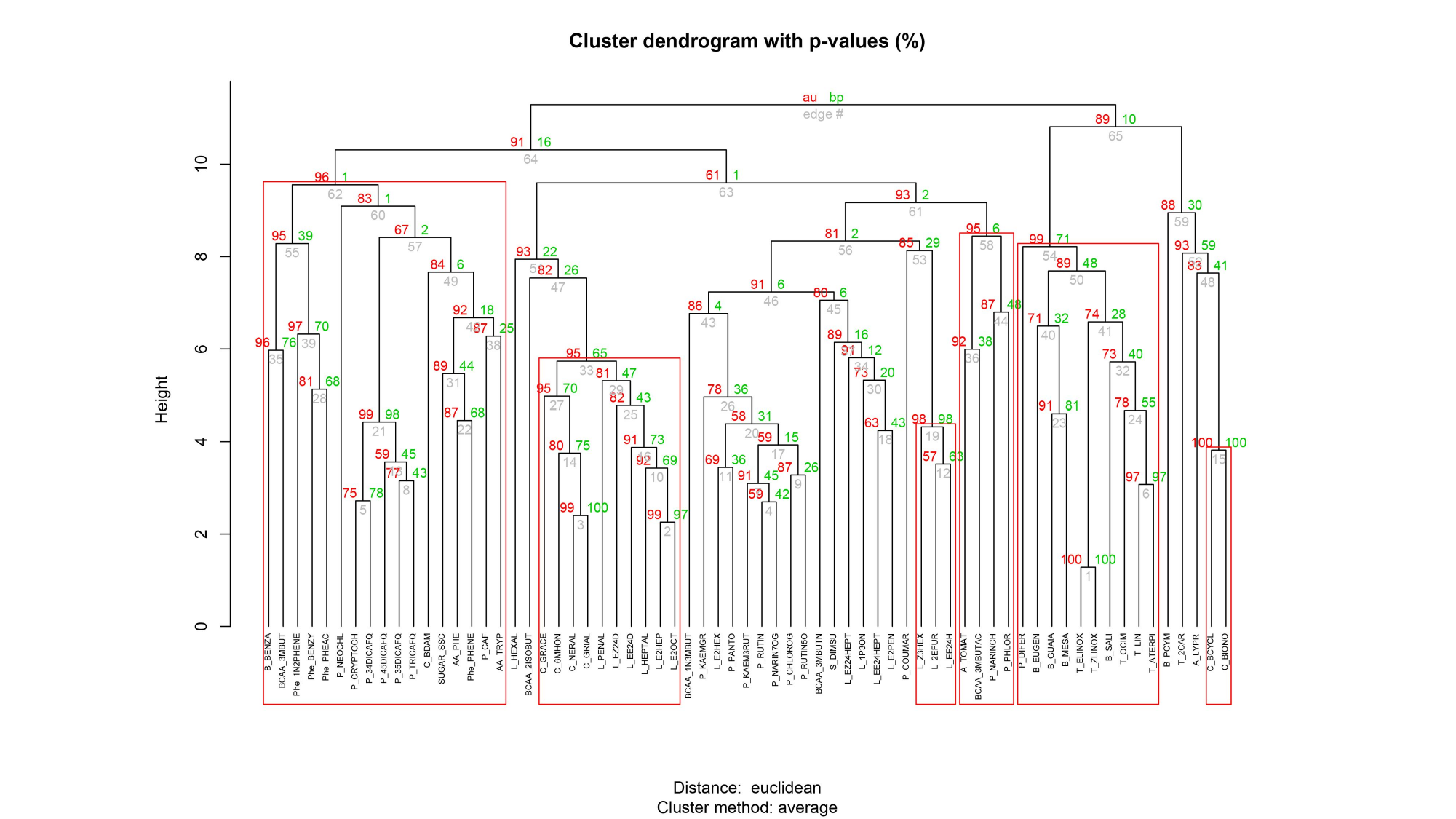

## Slide 4
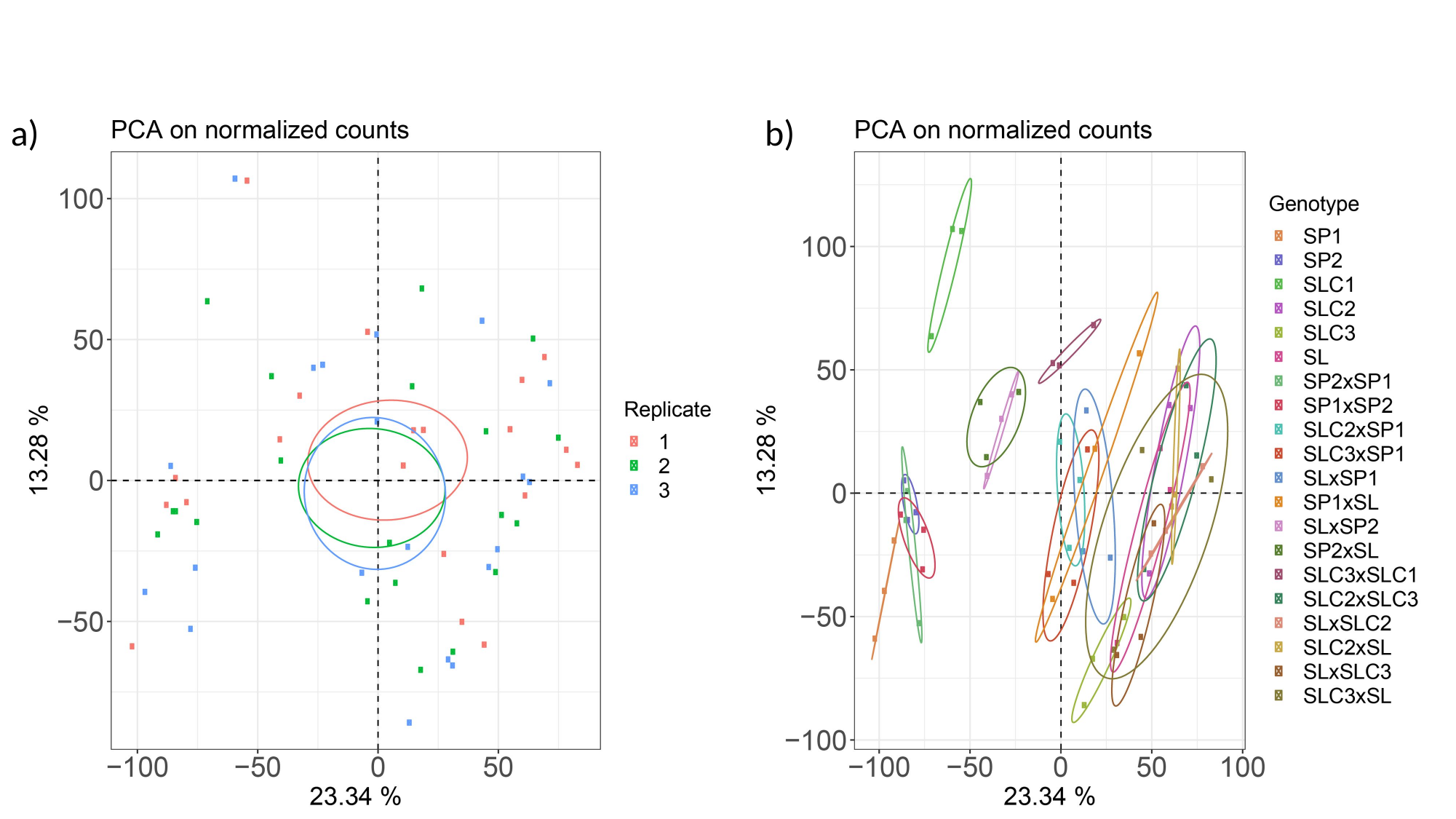

a)
b)

## Slide 5
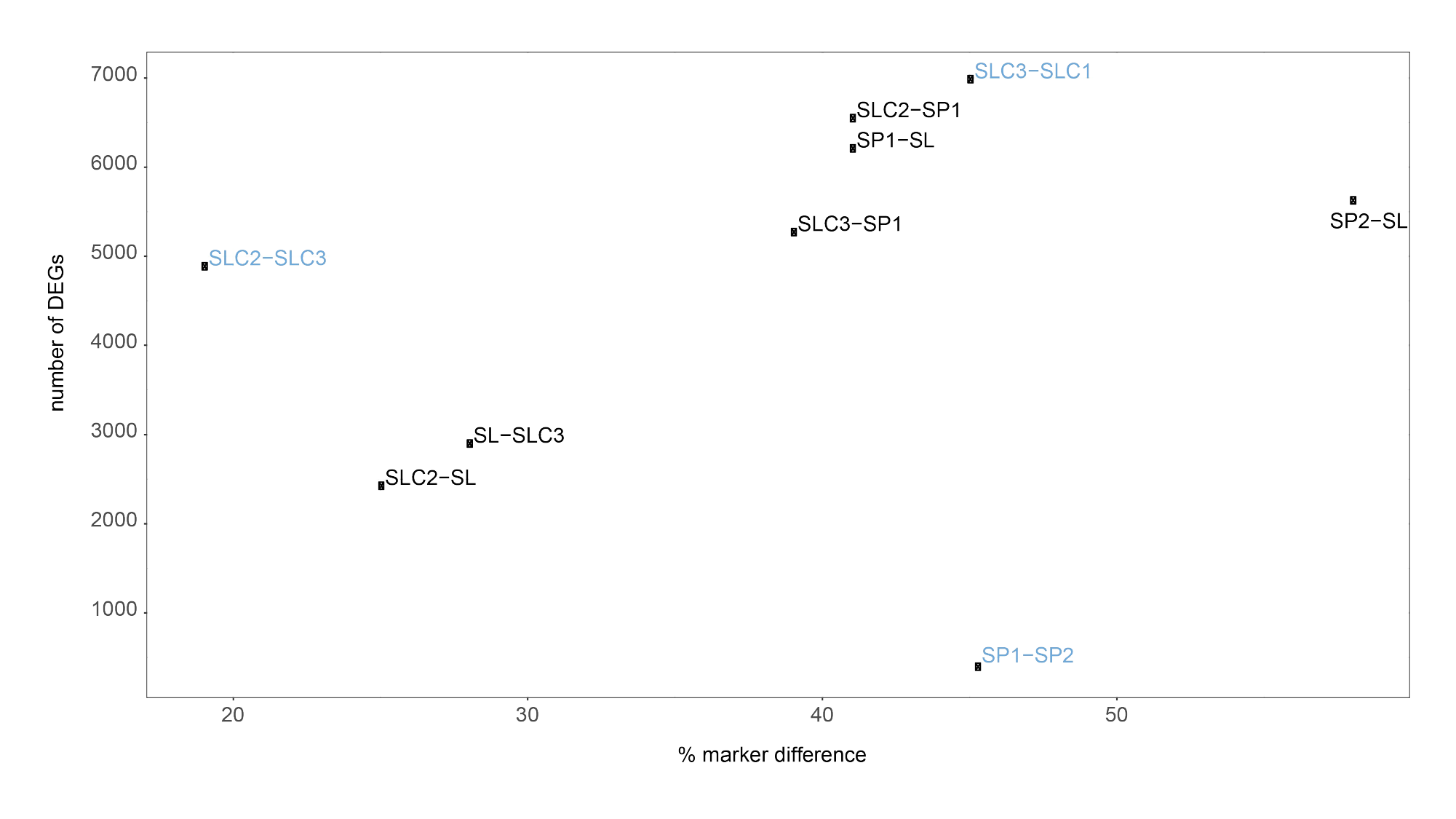

Supplement: Supplementary file 1 [file ijms-23-06163-s001.zip › Sup.Figures.pptx]
